# Potential repurposing of lapatinib and pazopanib as neuroprotective agents in a rat model of Huntington’s disease

**DOI:** 10.1007/s10787-025-01933-5

**Published:** 2025-09-10

**Authors:** Nada Ezeldine-Elmahalawy, Noha F. Abdelkader, Hala F. Zaki, Amany I. Elbrairy, Sameh S. Gad

**Affiliations:** 1 Postgraduate program in Pharmacology and Toxicology, Faculty of Pharmacy, Cairo University, Cairo, Egypt; 2 Department of Pharmacology, Faculty of Pharmacy, Cairo University, Cairo, Egypt; 3 Department of Medical Pharmacology, Faculty of Medicine, Cairo University, Giza, Egypt; 4 Department of Medical Pharmacology, Faculty of Dentistry, October University for Modern Sciences and Arts (MSA University), Giza, Egypt; 5 Department of Pharmacology and Toxicology, Faculty of Pharmacy, October University for Modern Sciences and Arts (MSA University), Giza, Egypt

**Keywords:** Huntington’s disease, Tyrosine kinase inhibitors, Lapatinib/pazopanib, NF-κB/TNF-α, Glutamate/calpain-2, PI3K/AKT/m-Tor/Beclin-1/LC3-II/ULK-1

## Abstract

The neuroprotective potential of tyrosine kinase inhibitors (TKIs), potent anticancer drugs, was verified against various neurodegenerative insults, but not Huntington’s disease (HD). These promising outcomes were due to their ability to modulate various intracellular signalling pathways. Hence, the current study aimed to evaluate the neuroprotective effects of lapatinib and pazopanib in the 3-nitropropionic (3-NP)-induced HD model in rats. After 14 days of 3-NP administration, rats received saline, lapatinib, or pazopanib for 21 days. Treatment with lapatinib or pazopanib improved the striatal microscopic architecture, neuronal survival, and neuroinflammatory responses, with a pronounced effect observed for pazopanib. At the molecular level, lapatinib and pazopanib reduced the striatal gene expression of NF-κB and TNF-α receptors, curbed the glutamate/calpain-2 axis, and modified the striatal content of inflammatory molecules as well as neurotransmitters. In addition, they activated the neuroprotective trajectory viz*.*, m-Tor/ULK-1/Beclin-1/LC3-II, an effect dependent on tyrosine kinase inhibition. Moreover, treated groups showed normalised tyrosine hydroxylase and glial fibrillary acidic protein in the striatum. In conclusion, this study provides strong evidence that lapatinib or pazopanib significantly improved motor function, alleviated cognitive decline, and attenuated neurodegeneration in HD rats via modulating key signalling pathways implicated in HD pathogenesis. These results underscore the promising therapeutic potential of TKIs in managing HD and warrant further investigation into their clinical application.

**Graphical abstract:**

Schematic representation summarising the mechanisms underlying the neuroprotective effects of Lapatinib and Pazopanib in 3-NP induced Huntington disease in rats. *LAP* Lapatinib, *PAZO* pazopanib, *ACh* acetylcholine, *NE* norepinephrine, *DA* dopamine, *5-HT* 5-hydroxytryptophan, *Glu* glutamate, *LC3* microtubule-associated protein 1A/1B-light chain, *ULK-1* Unc-51 like autophagy activating kinase, *m-Tor* mammalian target of rapamycin, *AKT* protein kinase B, *PI3K* phosphoinositide 3-kinase, *TNF-α* tumour necrosis alpha, *NFκB* nuclear factor kappa B, *GFAP* glial fibrillary acidic protein, *TH* tyrosine hydroxylase.

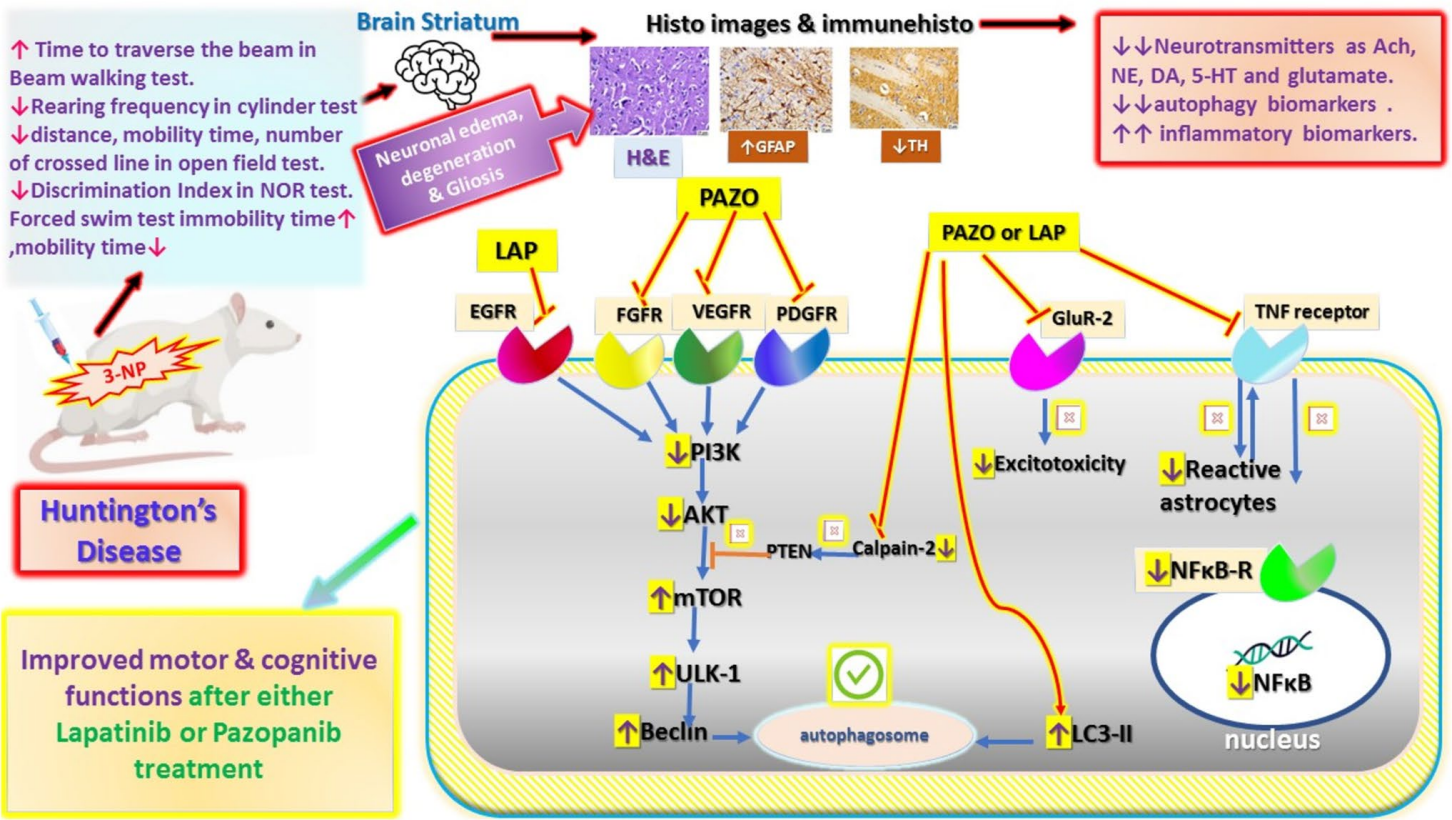

## Introduction

Huntington’s disease (HD) is a genetic neurodegenerative disorder characterised by progressive motor and cognitive impairment along with psychiatric disturbances. It has been reported that HD prevalence reached about 10 cases per 100,000 people, with higher rates reported in North America, northwestern Europe, the Middle East, and Australia (Crowell et al. 2021; Jiang et al. 2023). Moreover, a recent systematic review and meta-analysis revealed that the global prevalence and incidence of HD have been increased over the past decade due to de novo mutations, longer life expectancy, and advanced molecular diagnostics (Medina et al. 2022). Despite significant research progress, no disease-modifying therapy for HD currently exists; available treatments remain purely symptomatic and fail to halt disease progression.

The main factor in HD pathogenesis is a mutation that includes CAG repeat expansions in the huntingtin gene triggering the accumulation of mutant huntingtin protein (mHtt). This toxic protein causes severe degeneration in the cerebral cortex and striatum, leading to neurotransmitter imbalance and excitotoxicity (Zeron et al. 2002; Ross et al. 2014; Holland et al. 2021). Current HD therapies mainly focus on alleviating downstream effects like neuroinflammation and excitotoxicity (Jiang et al. 2023). Thus, there is a critical need for therapies that target upstream disease processes, particularly mHtt accumulation and associated cellular dysfunction. Notably, tyrosine kinase inhibitors (TKIs) including inhibitors of vascular endothelial growth factor receptor (VEGFR), platelet-derived growth factor receptor (PDGFR), and epidermal growth factor receptor (EGFR) promote clearance of neurotoxic proteins via autophagy, demonstrating neuroprotection in Parkinson’s disease (PD) and Alzheimer’s disease (AD) (Tavassoly et al. 2020). However, their therapeutic potential in HD remains unexplored.

Of note, autophagy is controlled by signalling pathways involving the mammalian target of rapamycin (m-Tor), protein kinase B (AKT), and phosphatidylinositol-3-kinase (PI3K). Dysregulation of the m-Tor pathway contributes to toxic protein accumulation, such as mHtt in HD, exacerbating neurodegeneration (Laplante and Sabatini 2012; Menon and Dhamija 2018; Esteves et al. 2019; Querfurth and Lee 2021). Therefore, modulating the PI3K/AKT/m-Tor pathway represents a promising therapeutic approach to clear neurotoxic protein aggregates and enhance neuronal survival (Nixon 2013; Li et al. 2020; De Marco et al. 2022).

The EGFR-TKIs, including afatinib, lapatinib (LAP), erlotinib, and gefitinib, improved memory and alleviated neuronal damage through the inhibition of EGFR intracellular signalling (Pulivarthi et al. 2023). Moreover, sunitinib, neratinib, and ponatinib exert neuroprotective effects associated with VEGFR and PDGFR signalling (Ryu et al. 2019; Dent et al. 2020; Liu et al. 2021; Kim et al. 2021). Likewise, pazopanib (PAZO) has been reported to reverse cognitive decline with a favourable safety profile in humans (Javidnia et al. 2017). Noteworthy, a recent clinical study displayed that nilotinib modulated autophagy and glial activity in HD patients though it did not affect mHtt and behavioural symptoms (Anderson et al. 2022), suggesting a promising mechanistic impact in HD. However, some TKIs like lorlatinib and vandetanib have been associated with neurotoxicity in non-small cell lung cancer patients and dopaminergic deterioration in PD preclinical study, respectively, emphasising the importance of careful selection and rigorous clinical validation of candidate TKIs (Requejo et al. 2018; Liu et al. 2021; Schoenmaekers et al. 2024).

The EGFR- and VEGFR-TKIs have demonstrated neuroprotective effects by inducing autophagy (Tavassoly et al. 2020), which might represent safe and effective approaches for managing neurodegenerative diseases. Amongst these, PAZO targets multiple tyrosine kinase receptors: VEGFR, PDGFR, fibroblast growth factor receptor (FGFR), and c-kit receptor (Javidnia et al. 2017). In addition, LAP, an EGFR-TKI and HER2-inhibitor, has been shown to modulate the abnormal PI3K/AKT/m-Tor signalling in HER2-positive breast cancer patients (Mansour et al. 2021a). Given their ability to cross the blood–brain barrier and target key molecular pathways that are involved in HD, LAP and PAZO may be promising candidates for repurposing as autophagy modulators to manage HD (Hingorani et al. 2014; Tavassoly et al. 2020). Therefore, the current investigation is designed to evaluate the possible neuroprotective effects of LAP and PAZO in an established experimental rat model of HD.

## Materials and methods

### Animals

Adult male Wistar rats (200–240 g) were purchased from the Nile Company for Pharmaceuticals & Chemical Industries, Cairo, Egypt. Rats were acclimatised in the animal facility of Faculty of Pharmacy, Cairo University, Cairo, Egypt. Animals were housed under standard conditions: A temperature of 25 ± 2 °C, 60 ± 10% humidity, and a 12/12-h light/dark cycle (lights on at 07:00 am) were maintained. Rats had unlimited access to standard chow pellets and water. All experimental procedures complied with the *Guide for Care and Use of Laboratory Animals* published by the US National Institutes of Health (NIH Publication No. 85-23, revised 2011) and the Ethics Committee for Animal Experimentation at Faculty of Pharmacy, Cairo University (Approval Number: PT 3107). All procedures were implemented to minimise animal suffering.

### Drugs and chemicals

3-Nitropropionic acid (3-NP; *PubChem* CID: 1678) was obtained from Sigma-Aldrich Co. (St Louis, MO, USA) and was prepared in normal saline (0.9% w/v) to a volume of 0.2 ml/200 g animal body weight for intraperitoneal injection (i.p.). Lapatinib ditosylate (*PubChem* CID: 208908) and pazopanib hydrochloride (*PubChem* CID: 11525740) were obtained from GlaxoSmithKline (Cairo, Egypt) and Novartis (Cairo, Egypt), respectively. Both drugs were prepared in normal saline (0.9% w/v) in a volume of 0.5 ml/200 g animal body weight for oral administration (p.o.). All other chemicals were of analytical grade.

### Experimental design

Sixty rats were randomly assigned to four groups (*n* = 15). The group sample size was determined by power analysis with statistical power of 0.8 and significance level (*α*) = 0.05, using the effect size evaluated earlier by Danduga et al. (2018). The group size was computed using the G Power software (version 3.1.9.4), incorporating effect size and attrition rate determined based on the results of the preliminary pilot study (data not shown).

**Fig. 1:**
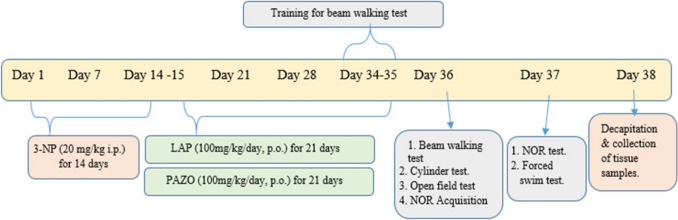
The experimental design. *3-NP* 3-nitropropionic acid, *LAP* lapatinib, *PAZO* pazopanib

Herein, animals in group 1 (normal) received saline. In group 2 (3-NP), animals received 3-NP (20 mg/kg/day, i.p.) for 14 days followed by 21 days of saline administration (Saad et al. 2021). In group 3 (3-NP + LAP) and group 4 (3-NP + PAZO), rats received 3-NP (20 mg/kg/day, i.p.) for 14 days, followed by daily oral administration of LAP (100 mg/kg/day) or PAZO (100 mg/kg/day), respectively, for 21 days (Wang et al. 2018; Mansour et al. 2021b). On days 34–35 and days 36–37, all animals were subjected to behavioural training followed by behavioural testing, respectively, according to the sequence illustrated in Fig. 1. One day after the behavioural analysis, animals in each group were randomly divided into two sets. Rats were euthanised by cervical dislocation after light ether anaesthesia. In the first set (*n* = 3), brains were immediately excised and fixed with 10% (v/v), neutral-buffered formalin for 24 h for histopathological and immunohistochemical staining. In the second set (*n* = 12), one striatum from each of six rats was promptly excised and stored at − 80 °C for molecular analysis via real-time PCR assay. The remaining striata were pooled in pairs with striata from the other six rats and kept at − 80 °C for subsequent evaluation of biochemical markers. An assistant who was not part of the research team performed the coding and decoding.

### Behavioural examination

#### Beam-walk test

Motor coordination in rats was assessed using the beam-walk test. A wooden rod (2.3 cm in diameter and 150 cm in length) was positioned 100 cm above the ground. To prevent injuries in the event of a fall, a cushion padded with foam was placed beneath the apparatus. Prior to data collection, rats were trained for 2 consecutive days. During testing, each rat was allowed 2 min to cross the beam with its home cage placed at the opposite end. Trials were cancelled if the animal took more than 2 min or fell off. Total crossing time was video recorded (Shivasharan et al. 2013).

#### Cylinder test

Since animals naturally use rearing behaviour to explore their environment, they were placed individually in a translucent plexiglass cylinder (30 cm in height, 20 cm in diameter) and were recorded for 5 min. The total number of rearing was assessed to evaluate the forepaws’ function and musculoskeletal coordination. A rear was identified when the rat lifted one or both forelimbs higher than shoulder level and touched the cylinder wall. A second rear was only counted when the rat withdrew its forepaws and touched the floor again. ANY-maze video-tracking system was used for scoring (Version 0.6.3.3, Stoelting Co., Wood Dale, IL, USA) (Fleming et al. 2004; El-Abhar et al. 2018).

#### Open-field test

This test was employed to assess locomotion in rodents. Each rat was individually located centrally within a wooden box (80 × 80 × 40 cm) with red walls and a black floor, subdivided into 16 squares with white margins. Rats were permitted to discover the arena freely and were filmed for 5 min. Cleaning with 70% ethanol was conducted following each rat. ANY-maze video-tracking system was used to assess total distance, average speed, time mobile, immobile as well as number of crossed lines (Version 0.6.3.3, Stoelting Co., Wood Dale, IL, USA) (Sayed et al. 2020; El-Kadi et al. 2025).

#### Novel object recognition test

Assessment of short-term recognition memory for rats was conducted by measuring the time spent by the animals to discover a novel object compared to a familiar one. The device consisted of a wooden box (65 × 45 × 65 cm). A habituation phase was conducted, where each rat was allowed a 5-min session to explore the testing arena with no objects placed. On the next day, a familiarisation phase was conducted where each rat was individually located in the testing arena with two identical objects (A1 & A2, green wooden cubes) and was allowed to explore the objects for 5 min. After 1 h, each rat was placed in the testing arena with one of the familiar objects (A) and a novel object (B, yellow wooden cylinder) and was allowed 5 min to explore the objects as a testing phase to assess the short-term recognition memory. Exploration was considered when the rat sniffed or touched the object with its nose or forelimbs, respectively; sitting on the object was not classified as exploration (Velloso et al. 2009). Between trials, the objects were cleaned with 70% ethanol solution to prevent bias. The time spent touching or sniffing the objects was recorded using ANY-maze video-tracking software (Version 0.6.3.3, Stoelting Co., Wood Dale, IL, USA). Discrimination index (DI) was evaluated using the equation TB/(TA + TB), where TA represents the time spent exploring the familiar object and TB represents the time spent exploring the novel one (Pietá Dias et al. 2007).

#### Forced swim test

The forced swim test was applied to measure the depressive-like behaviour of rats. Each rat was placed individually in a transparent plexiglass cylindrical tank (50 cm high, 20 cm diameter), which was filled with water to a depth of 40 cm and maintained at 25 ± 2 °C. After a 1-min acclimation period, the immobility time was recorded over the remaining 5 min. An immobile animal was defined when it stopped struggling, making only the essential movements to keep its head above water. Following each trial, the tank was washed and refilled with fresh water (Kamal et al. 2025). Time mobile or immobile was recorded using ANY-maze video-tracking software (Version 0.6.3.3, Stoelting Co., Wood Dale, IL, USA).

### Biochemical analysis

#### Enzyme-linked immunosorbent assay

Quantification of striatal PI3K, Glu, Beclin-1, NE, ACh, DA, and NF-κB was conducted using ELISA kits from MyBioSource, San Diego, CA, USA (Cat. No. #MBS260381, MBS756400, MBS901662, MBS269993, MBS282680, MBS725908, MBS453975, respectively). In addition, TNF-α (Cloud-Clone Corp., Katy, TX77494, USA; Cat. No. #SEA133Ra), ULK-1 (Assaygenie; Cat. No. #RTFI01416), m-Tor'zs and AKT (LSBIO, Seattle, WA, USA; Cat. No. #LS-F17553, Cat. No. #LS-F1447), LC3-II (CELL BIOLABS, INC., San Diego, CA; Cat. No. #CBA-5116), calpain-2 (Antibodies-online GmbH, Aachen, Germany; Cat. No. #ABIN6954298), and 5-HT (Abcam, Cambridge, UK; catalogue no. #ab133053) were evaluated as per the manufacturers’ guidelines. The results were expressed as ng/mg protein for PI3K, AKT, mTOR, Beclin-1, NF-κB, LC3-II, calpain-2, 5-HT, DA and as pg/mg protein for ULK-1, TNF-α, ACh, and NE. Glu was expressed as nmol/mg protein. Total protein content in the tissue homogenates was determined following the Lowry method for protein quantification (Waterborg and Matthews 2003).

#### Quantitative real-time PCR (qRT-PCR) assay

The gene expression levels of TNF-α and NF-κB receptors were assessed. In accordance with the manufacturer’s instructions, total RNA was extracted from tissue lysates with Direct-zol RNA Miniprep Plus (Cat. No. R2072, Zymo Research Corp., USA). Subsequently, 0.5–2 μg of the isolated RNA was reverse-transcribed into cDNA using SuperScript IV One-Step RT-PCR kit (Cat. No. 12594100, Thermo Fisher Scientific, Waltham, MA, USA). Total synthesised cDNA was amplified and analysed using Applied Biosystems (StepOne™, software version 3.1, USA) as per the manufacturer’s guidelines. The calculation of relative quantification (RQ) of mRNA of TNF-α receptor and NF-κB receptor was determined using the formula expressed by the software: RQ = 2^−ΔΔCT^, where ΔCT = Ct_gene test − Ct_endogenous control (GAPDH) and ΔΔCt = ΔCt_sample1 − ΔCt_calibrator. Results were normalised to GAPDH and expressed as arbitrary units. The primer sequence used in the annealing phase is listed in Table 1.

**Table 1:** The sequence of primers used in quantitative real-time polymerase chain reaction (qRT-PCR) in the annealing phase

Gene	Primer sequence	
TNF-α receptor	Forward	TAGCTCCCAGAAAAGCAAGC
	Reverse	TTTTCTGGAGGGAGATGTGG
NF-κB receptor	Forward	CATGAAGAGAAGACACTGACCATGGAAA
	Reverse	TGGATAGAGGCTAAGTGTAGACACG
GAPDH	Forward	TGGATTTGGACGCATTGGTC
	Reverse	TTTGCACTGGTACGTGTTGAT

### Histopathological examination

Brains of rats were dissected out and fixed in neutral-buffered formalin (10%), they were subjected to routine preparation through graded alcohols and xylene, followed by paraffin embedding and cutting into 5 µm sections for haematoxylin and eosin staining (H&E) (Bancroft and Gamble 2007). A scoring system was used to evaluate the detected histopathological changes, including oedema, neuronal degeneration, and gliosis, on a scale ranging from 1 to 4 according to the severity of the lesion. Darkly degenerated neurons were quantified across five non-overlapping histological fields at 400× magnification inside a standard measuring frame of 85,550 µm^2^ using the ImageJ analysis system (J Image Pro Plus 6.0, Media Cybernetics, Silver Spring, MD, USA). Digital images were consistently captured by an Olympus IX51 microscope equipped with a DP72 camera.

### Immunohistochemical examination

Quantification of tyrosine hydroxylase (TH) and glial fibrillary acidic protein (GFAP) was achieved using paraffin-fixed striatal sections. Tissue sections of 5 µm thickness were sliced for immunostaining. Striatal sections were processed by heat retrieval step followed by blocking of endogenous peroxidase activity. Sections were incubated with primary anti-GFAP and anti-TH (at a dilution of 1:150 and 1:1000, respectively) for 2 h at room temperature. After washing, Mouse/Rabbit Immuno-Detector DAB-HRP kit (Bi SB, CA, USA) was utilised in accordance with manufacturer’s guidelines to develop the brown colour of positive reaction. Negative control slides were prepared by deletion of primary antibody step. Positive expression was calculated as area per cent using CellSens dimensions Olympus software (Olympus, Tokyo, Japan). Slides were subsequently counterstained using haematoxylin, dehydrated, coverslipped, and examined under a light microscope. The number of GFAP and TH immunoreactive cells in a unit area was then quantified. The immunoreactive cells per mm² in each rat brain were expressed as the mean of the three individual sections. Immunoreactivity was predominantly cytoplasmic and graded as: negative (−), mild (+), moderate (++), and marked (+++) (Abdelkader et al. 2025). In addition, the count of TH positive neurons per each rat brain section was assessed in 3 non-overlapping high-magnification fields, and the results were statistically analysed.

### Statistical analysis

The data are expressed as mean ± standard deviation (S.D.). Statistical analysis is conducted by one-way ANOVA and *Tukey–Kramer’s* post hoc test with significance considered at *p* < 0.05 for all statistical tests. The histopathological scoring is reported as median and range and is analysed using *Kruskal–Wallis* non-parametric one-way ANOVA and Dunn’s post hoc test. The correlation coefficient is evaluated by linear regression analysis. All statistical analysis and graphical representations are accomplished using GraphPad Prism software (version 8; GraphPad Software, Inc., San Diego, CA, USA).

## Results

### Effect of lapatinib and pazopanib on motor activity and coordination

**Fig. 2:**
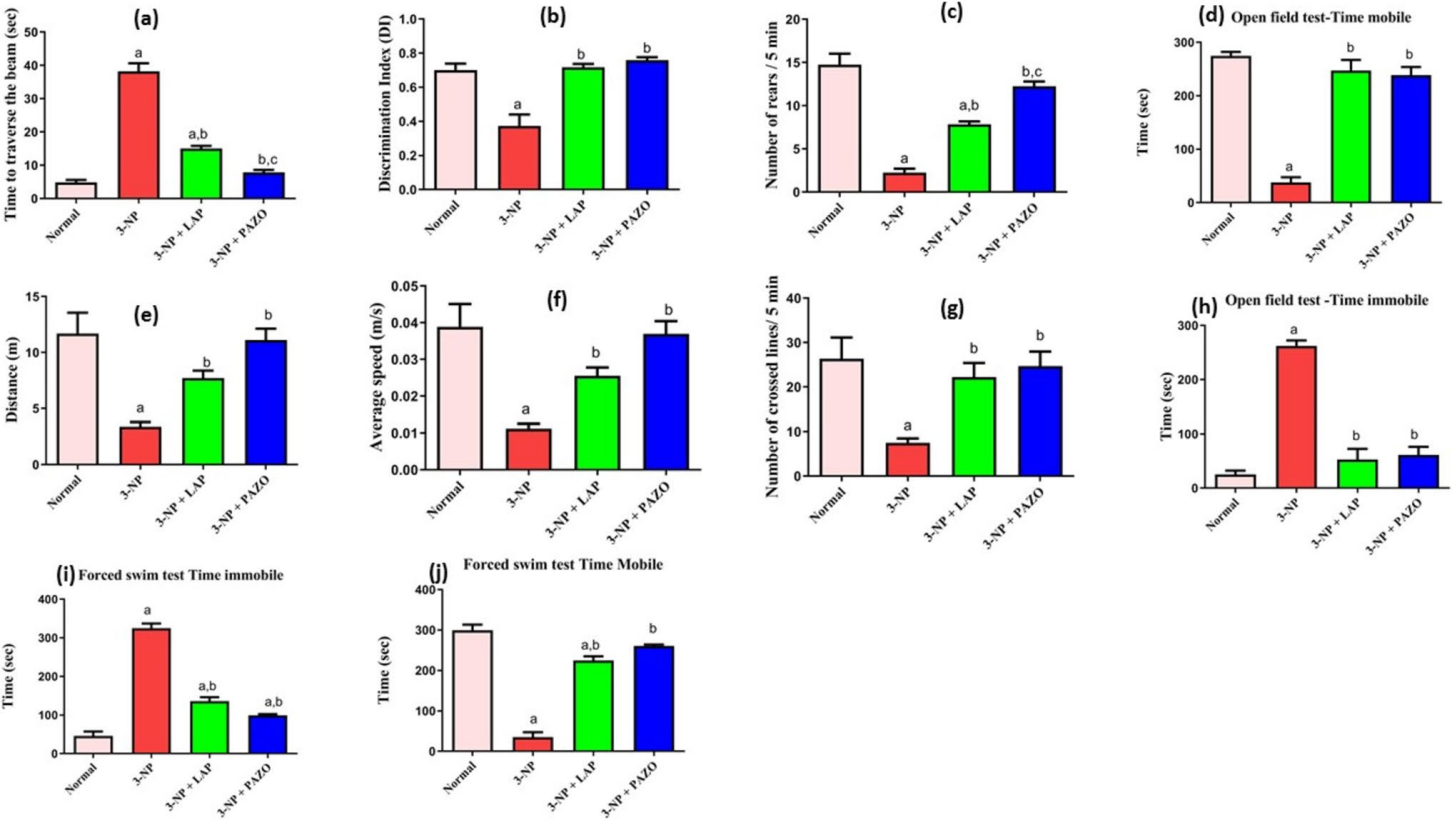
Effect of lapatinib and pazopanib on the 3-NP-induced motor and cognitive impairment and psychiatric disturbances. **a** Time to traverse the beam, **b** discrimination index, **c** number of rears, **d** open field time mobile, **e** distance, **f** average speed, **g** number of crossed lines, **h** open field time immobile, **i** forced swim test immobility time, and **j** forced swim mobility time. Data are presented as mean ± S.D. (*n* = 12) using a bar graph with error bars. ^a^vs normal group, ^b^vs 3-NP group, ^c^vs 3-NP + LAP. Statistical significance was observed in all measured parameters at *p* < 0.05. *3-NP* 3-nitropropionic acid, *LAP* lapatinib, *PAZO* pazopanib

Herein, intoxication with 3-NP prolonged the time required to traverse the beam in the beam-walking test to 7.9-fold, whilst it diminished the total number of rears during the cylinder test by 84.7% relative to the normal group (Fig. 2a, c). Moreover, 3-NP administration reduced the total distance that rats travelled in the open field test by 71.8% and delayed their average speed to 29% relative to the normal group. In addition, exposure to 3-NP increased the time immobile in the open field test by 9.4-fold, whilst reducing mobile time by 86% and the number of crossed lines by 53% relative to the normal group (Fig. 2d–h). Conversely, in the LAP- and PAZO-treated groups, improvements in motor behaviour were observed as demonstrated by diminished time to traverse the beam and an acceleration of 60.7% and 79.5%, respectively, in comparison with the 3-NP group. Furthermore, an improvement in rearing frequency in the cylinder test was observed as LAP and PAZO increased it by 3.5- and 5.4-fold, respectively. In the open field test, animals treated with LAP and PAZO revealed a significant upgrade in distance to 2.2- and 3.7-fold, besides average speed to 2.3- and 2.7-fold compared to the 3-NP group, respectively. They also expanded the number of crossed lines by 2- and 2.3-fold relative to the 3-NP group. Administration of LAP and PAZO decreased the immobile duration by approximately 80% and 77% and increased the mobile time by 6.5- and 6.3-fold relative to the 3-NP group.

### Effect of lapatinib and pazopanib on cognition and psychiatric behaviour

Herein, 3-NP administration prolonged immobility time in the forced swim test by almost sevenfold and decreased mobile time by 88%, whereas it reduced the discrimination index in the novel object recognition test by 47% relative to the normal group (Fig. 2b, i, j). Treatment with LAP and PAZO halved the immobility time relative to the observed values of the 3-NP group and enhanced the mobile time by 6.4- and 7.5-fold, respectively. Moreover, LAP and PAZO treated animals presented an increase in the discrimination index by 192% and 203.6%, respectively, relative to the 3-NP group.

### Effect of lapatinib and pazopanib on striatal neurotransmitters

**Fig. 3:**
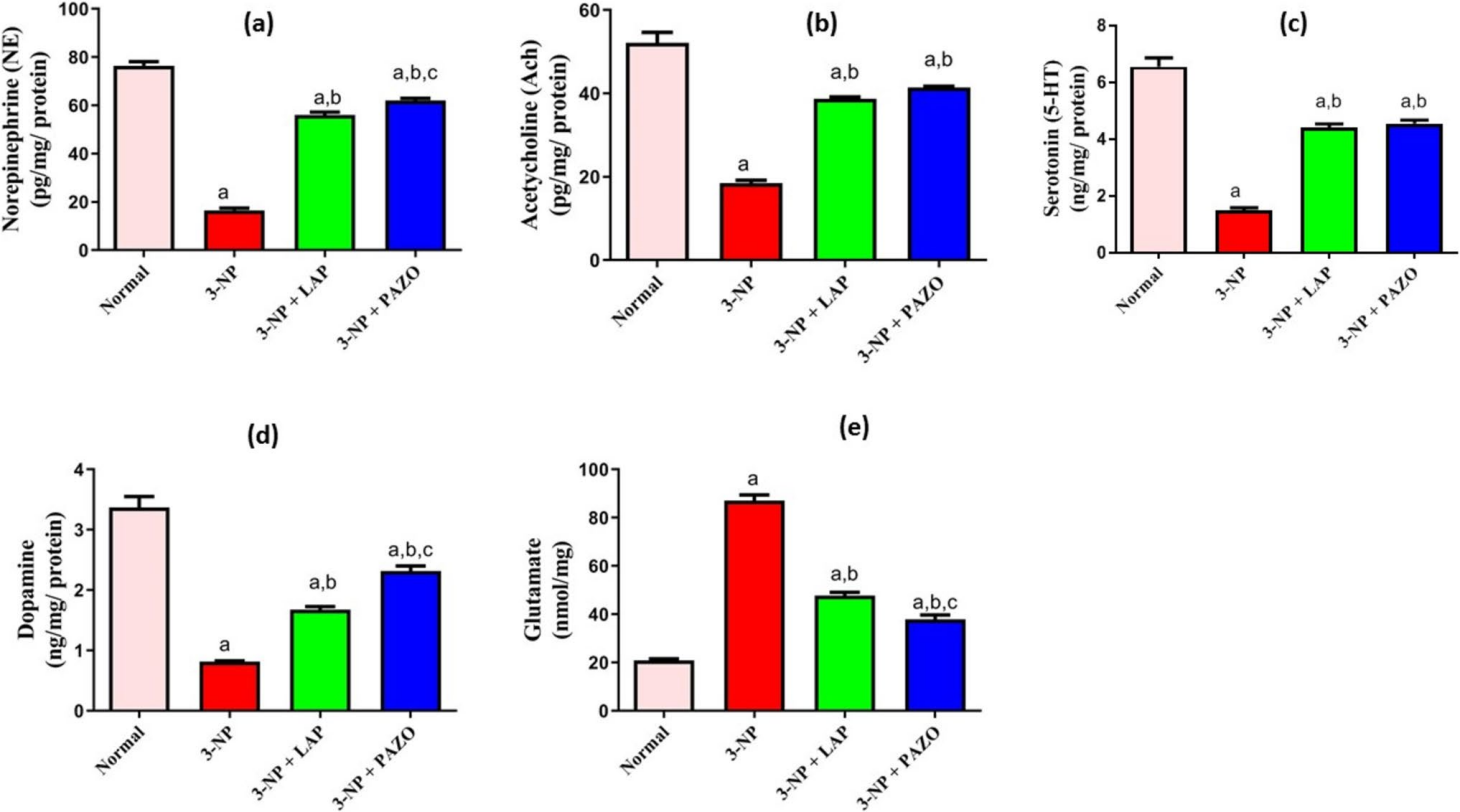
Effect of lapatinib and pazopanib on the 3-NP-induced changes in striatal neurotransmitters. **a** NE, **b** Ach, **c** 5-HT, **d** DA, and **e** glutamate. Data are presented as mean ± S.D. (*n* = 6) using a bar graph with error bars ^a^vs normal group, ^b^vs 3-NP group, ^c^vs 3-NP + LAP. Statistical significance was observed in all measured parameters at *p* < 0.05. *3-NP* 3-nitropropionic acid, *LAP* lapatinib, *PAZO* pazopanib

Following 3-NP administration, ACh, NE, DA, and 5-HT were decreased to approximately 75% of the normal values, whereas Glu was elevated by 4.2-fold (Fig. 3). LAP treatment significantly attenuated the reduction in ACh, NE, DA, and 5-HT and increased them by 2-, 3.4-, 2-, and threefold, respectively, relative to the 3-NP group. Similarly, PAZO treatment significantly inhibited the reduction in ACh, NE, DA, and 5-HT and increased them by 2.24-, 3.74-, 2.85-, and threefold, respectively, in comparison with the 3-NP group. Both LAP and PAZO treatments decreased Glu levels by 55% and 43.5%, respectively, relative to the 3-NP group. Furthermore, PAZO administration increased NE and DA levels by 7.8% and 7.9%, respectively, and decreased Glu levels by 11.4% as compared to LAP treatment.

### Effect of lapatinib and pazopanib on striatal autophagy related mediators

**Fig. 4:**
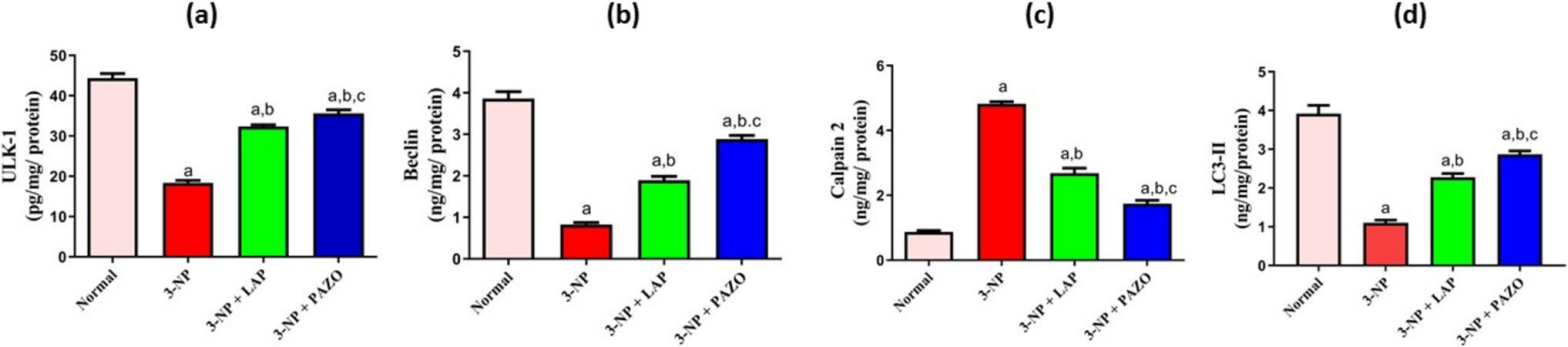
Effect of lapatinib and pazopanib on the 3-NP-induced alterations in striatal autophagy related proteins. **a** ULK-1, **b** beclin-1, **c** calpain-2, and **d** LC3-II contents. Data are presented as mean ± S.D. (*n* = 6) using a bar graph with error bars. ^a^vs normal group, ^b^vs 3-NP group, ^c^vs 3-NP + LAP. Statistical significance was observed in all measured parameters at *p* < 0.05. *3-NP* 3-nitropropionic acid, *LAP* lapatinib, *PAZO* pazopanib, *LC3* microtubule-associated protein 1A/1B-light chain, *ULK-1* Unc-51 like autophagy activating kinase

As demonstrated in Fig. 4, 3-NP injection markedly diminished ULK-1, Beclin-1, and LC3-II levels by nearly 58.4%, 78%, and 71.8% respectively, whilst it raised calpain-2 by 5.5-fold relative to the normal group. Administration of LAP increased ULK-1, Beclin-1, and LC3-II by 1.76-, 2.28-, and 2.36-fold, respectively, relative to the 3-NP group. Similarly, PAZO treatment heightened ULK-1, Beclin-1, and LC3-II by 1.93-, 3.47-, and 2.6-fold, respectively, relative to the 3-NP group. Moreover, administration of LAP and PAZO markedly reduced calpain-2 by 44.39% and 64%, respectively, relative to the values of the 3-NP group. Notably, PAZO enhanced ULK-1, Beclin-1, and LC3-II by 7.2%, 27.2%, and 15.1% respectively, whilst it decreased calpain-2 by 19.6%, in comparison with LAP treatment.

### Effect of lapatinib and pazopanib on striatal PI3K/AKT/m-Tor signalling

**Fig. 5:**
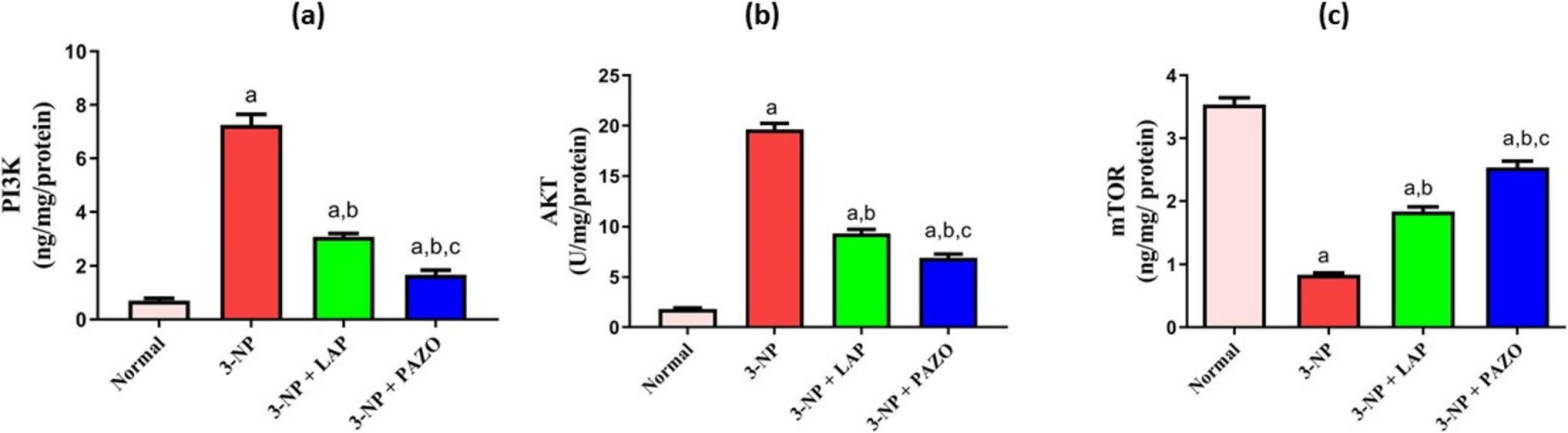
Effect of lapatinib and pazopanib on the 3-NP-induced alterations in striatal m-Tor-dependent autophagy pathway. **a** PI3K, **b** AKT, and **c** m-Tor contents. Data are presented as mean ± S.D. (*n* = 6) using a bar graph with error bars. ^a^vs normal group, ^b^vs 3-NP group, ^c^vs 3-NP + LAP. Statistical significance was observed in all measured parameters at *p* < 0.05. *3-NP* 3-nitropropionic acid, *LAP* lapatinib, *PAZO* pazopanib, *m-Tor* mammalian target of rapamycin, *AKT* protein kinase B, *PI3K* phosphoinositide 3-kinase

As illustrated in Fig. 5, the 3-NP group exhibited a 76% decrease in m-Tor and a marked rise in AKT and PI3K levels by 10.8- and 10.2-fold, respectively, relative to the normal group. LAP and PAZO administration increased m-Tor expression by 2.29- and threefold, respectively, whilst reducing AKT levels by 52.5% and 65%, and PI3K levels by 57.4% and 76.8%, compared to the 3-NP group. Moreover, PAZO treatment increased m-Tor levels by 21.2% and decreased AKT and PI3K by 12.5% and 19.5%, respectively, as compared to LAP treatment.

### Effect of lapatinib and pazopanib on striatal inflammatory mediators

**Fig. 6:**
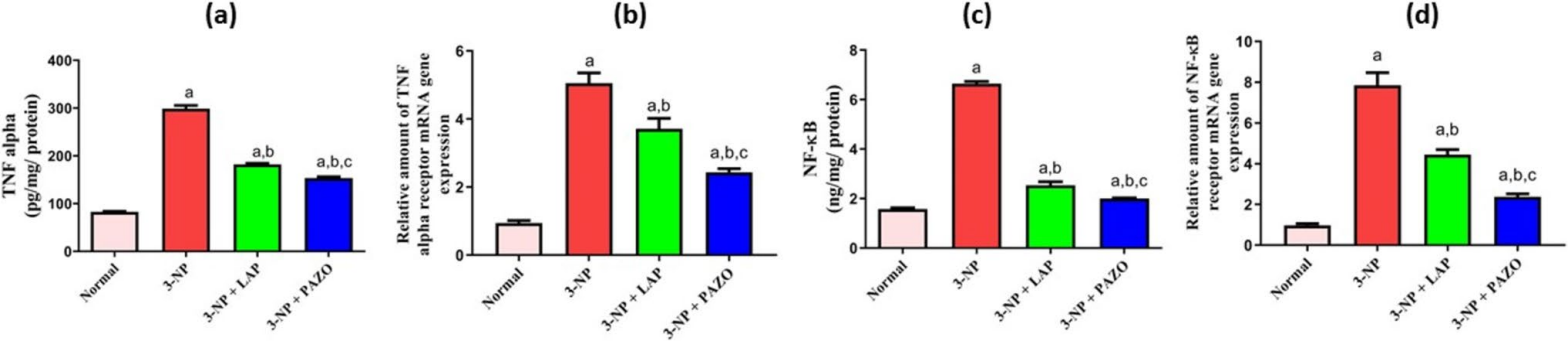
Effect of lapatinib and pazopanib on the 3-NP-induced alterations in striatal inflammatory pathway. **a** TNF-α content, **b** TNFα receptor expression, **c** NF-κB content, **d** NF-κB receptor expression. Data are presented as mean ± S.D. (*n* = 6) using a bar graph with error bars. ^a^vs normal group, ^b^vs 3-NP group, ^c^vs 3-NP + LAP. Statistical significance was observed in all measured parameters at *p* < 0.05. *3-NP* 3-nitropropionic acid, *LAP* lapatinib, *PAZO* pazopanib, *TNF-α* tumour necrosis alpha, *NFκB* nuclear factor kappa B

**Fig. 7:**
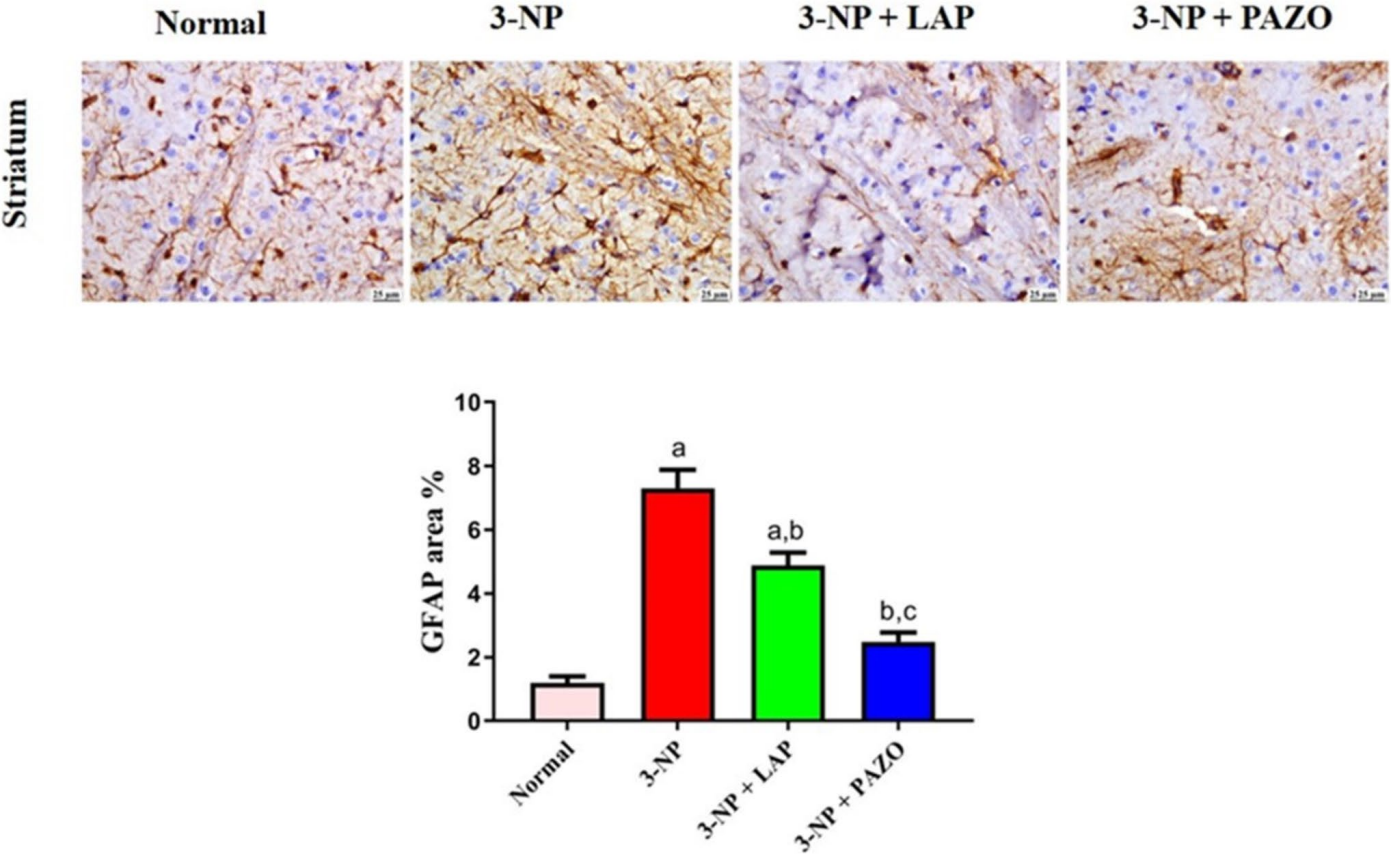
Photomicrographs of striatal sections showing GFAP immunoexpression in the different experimental groups. The chart represents quantification of GFAP expression (as area per cent). Data are presented as mean ± S.D. (*n* = 3) using a bar graph with error bars. ^a^vs normal group, ^b^vs 3-NP group, ^c^vs 3-NP + LAP. Statistical significance was observed in all measured parameters at *p* < 0.05. *3-NP* 3-nitropropionic acid, *LAP* lapatinib, *PAZO* pazopanib

In Fig. 6, the 3-NP group showed a 3.6- and 4.4-fold rise in TNF-α and NF-κB, respectively. In parallel, a considerable increase in the gene expression of TNF-α and NF-κB receptors by 5.6- and eightfold, respectively, was observed relative to the normal rats. Moreover, GFAP immunoexpression in the 3-NP group was increased by 6.1-fold relative to the normal group, which showed normal limited GFAP expression in striatal tissues (Fig. 7). On the other hand, treatment with LAP and PAZO reduced TNF-α by 39% and 48.6% and NF-κB by 62% and 69.7%, respectively, compared to the 3-NP group. Moreover, LAP diminished the striatal gene expression of TNF-α and NF-κB receptors by 26.7% and 43.5% of the 3-NP group, respectively. Also, PAZO diminished the striatal gene expression of TNF-α and NF-κB receptors by 52.47% and 70.5% relative to the 3-NP group, respectively. Administration of LAP and PAZO diminished GFAP positive expression by 33.2% and 66%, respectively, relative to the 3-NP group. Notably, relative to LAP treatment, PAZO treatment decreased striatal TNF-α and NF-κB levels, TNF-α and NF-κB receptors’ expression, and GFAP by 9.6%, 7.7%, 25.8%, 27%, and 33%, respectively, as compared to LAP treatment.

### Effect of lapatinib and pazopanib on striatal histopathological alterations

**Fig. 8:**
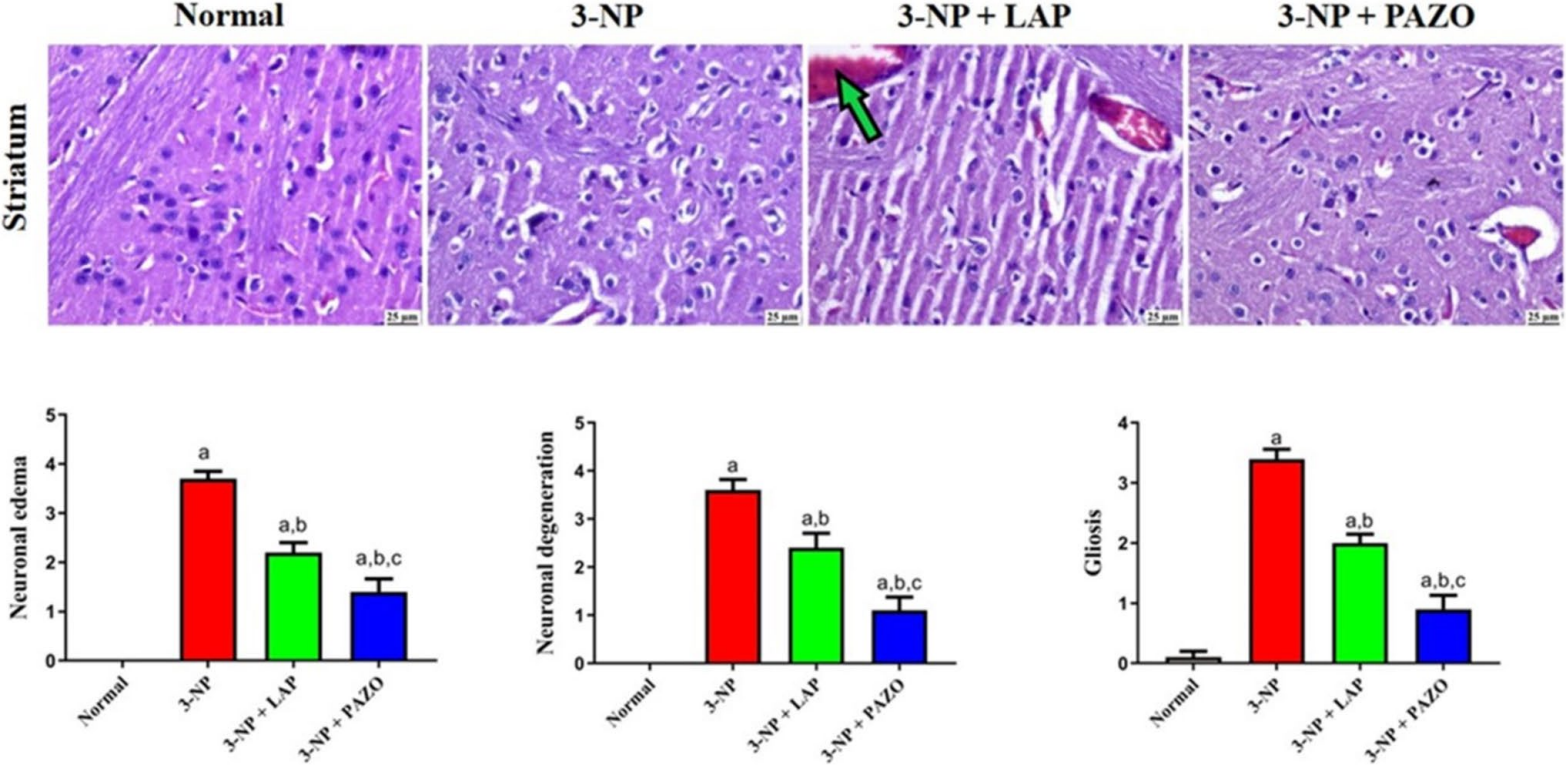
Photomicrographs of striatal sections in the different experimental groups. H&E-stained sections showing normal brain structure in normal saline group, area of Malacia, neuronal oedema, and vasculitis in 3-NP group. Some dark neurons and congestion (green arrows) in 3-NP + Lapatinib. Apparently normal brain regions in 3-NP + Pazopanib group. Charts of histological score. Data are presented as mean ± S.D. (*n* = 3) using a bar graph with error bars. ^a^vs normal group, ^b^vs 3-NP group, ^c^vs 3-NP + LAP. Statistical significance was observed in all measured parameters at *p* < 0.05. *3-NP* 3-nitropropionic acid, *LAP* lapatinib, *PAZO* pazopanib (Color figure online)

The normal group exhibited normal histological structure of the striatum upon microscopic examination (Fig. 8). By contrast, the 3-NP group demonstrated marked histopathological changes, including focal areas of malacia with neuronal necrosis and an intense glial reaction. Administration of 3-NP increased gliosis, neuronal oedema, and neuronal degeneration by 3.4-, 3.7-, and 3.6-fold, respectively, in comparison to the normal group. Treatment with LAP alleviated the neuronal damage as evidenced by the presence of few dark, degenerating neurons with neuronophagia in few sections; alongside, mildly congested blood vessels and mild neuronal oedema were observed. The observed gliosis, neuronal oedema, and neuronal degenerations were decreased by 41.2%, 40.5%, and 33.3%, respectively, in comparison to the 3-NP group. In the PAZO-treated group, the striatum appeared normal, without detectable histopathological alterations, and demonstrated markedly decreased gliosis, neuronal oedema, and neuronal degenerations by 73.5%, 62.2%, and 69.4%, respectively, compared with the 3-NP group and by 32.3%, 21.6%, and 36.1%, respectively, relative to the 3-NP + LAP group.

### Effect of lapatinib and pazopanib on striatal tyrosine hydroxylase

**Fig. 9:**
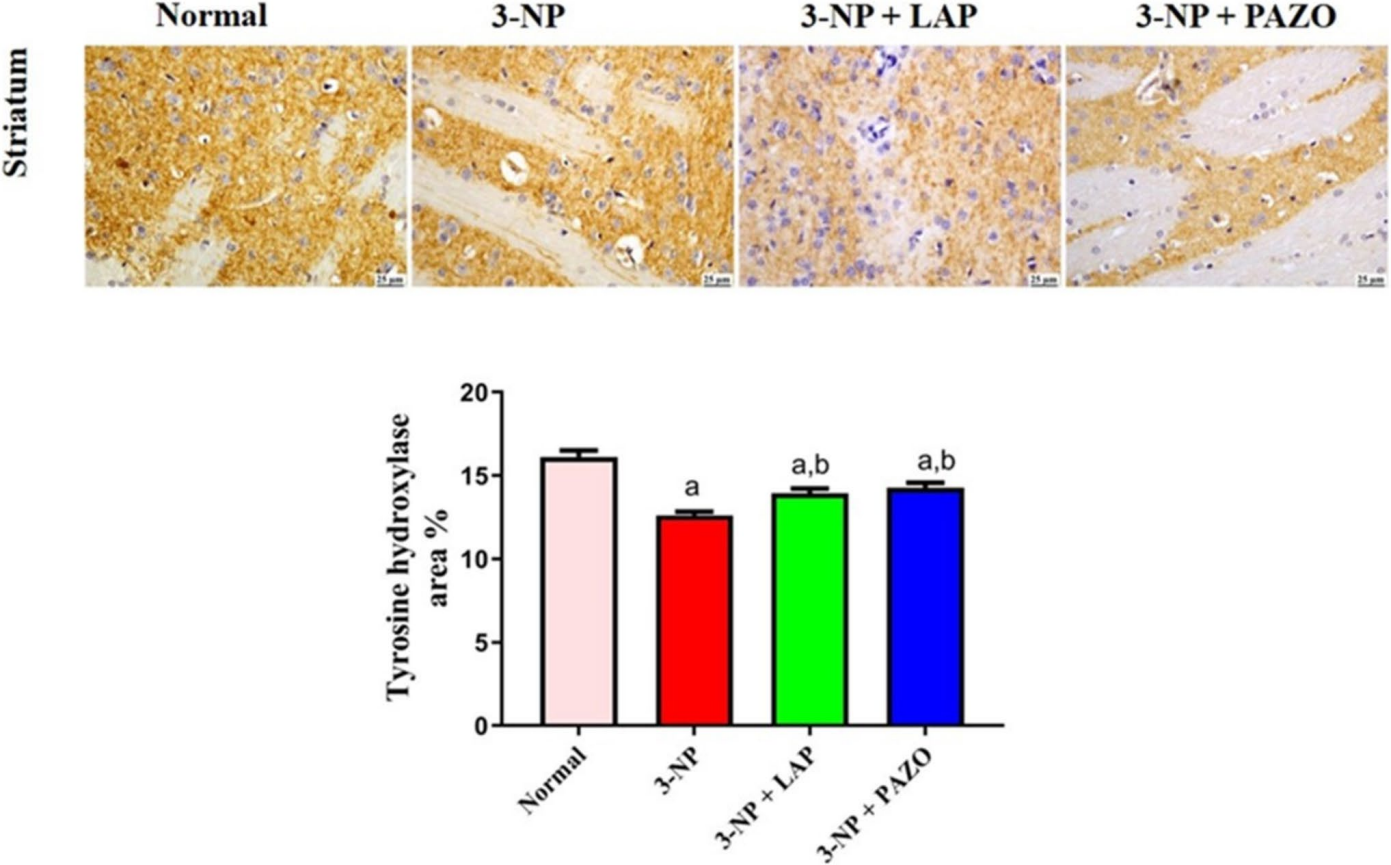
Photomicrographs of striatal sections showing TH immunoexpression in the different experimental groups. Chart represents TH expression quantification (as area per cent). Data are presented as mean ± S.D. (*n* = 3) using a bar graph with error bars. ^a^vs normal group, ^b^vs 3-NP group, ^c^vs 3-NP + LAP. Statistical significance was observed in all measured parameters at *p* < 0.05. *3-NP* 3-nitropropionic acid, *LAP* lapatinib, *PAZO* pazopanib

The 3-NP group demonstrated a marked reduction in striatal TH by 21.8% as compared to the normal group (Fig. 9). In contrast, both treatment groups 3-NP + LAP and 3-NP + PAZO exhibited significant increases in TH-positive staining by 10.5% and 13.2%, respectively, as compared to the 3-NP group.

## Discussion

The current findings reveal a novel repurposing of LAP and PAZO as potential candidates for HD treatment, with a pronounced effect observed for PAZO. These TKIs showed neuroprotective effects, counteracting the neurobehavioral deficits of the 3-NP induced HD-like symptoms in rats via modulating the inflammatory and PI3K/AKT/m-TOR-mediated autophagy signalling.

In the current study, intoxication with 3-NP impaired locomotor performance as evidenced by prolonged time to traverse the beam in the beam-walking test, decreased total number of rears in the cylinder test, and decreased distance travelled, average speed, mobile time, and number of crossed lines alongside increased immobile time in the open field test. In addition, a marked memory regression and mood instability were confirmed by a reduced DI in the novel object recognition test and prolonged immobility time in the forced swim test. Such behavioural abnormalities are consistent with previous reports (Kumar et al. 2006; Suganya and Sumathi 2017; Saad et al. 2021), which could be explained by severe neurodegeneration of intrinsic striatal neurons following 3-NP intoxication, leading to progressive dementia and involuntary abnormal choreiform movements (Borlongan et al. 1997). Conversely, treatment with LAP or PAZO exhibited a significant improvement in the locomotor, cognitive, and psychiatric behaviour, as manifested by short time to traverse the beam and high rearing frequency (cylinder test), increased distance, average speed, and number of lines crossed (open field test), short immobility time (forced swim test), and enhanced DI (novel object recognition test). These findings align with prior evidence of several TKIs benefits in preclinical studies of various neurological disorders including AD, PD, HD, and amyotrophic lateral sclerosis (Le Pichon et al. 2013; Pagan et al. 2016; Mansour et al. 2021b; Li et al. 2022). Notably, the PAZO group exhibited significantly greater rearing frequency and a decline in the time to traverse the beam when compared to the LAP group, possibly reflecting its broader inhibitory action on multiple tyrosine kinase receptors, including FGFR, VEGFR, PDGFR, rather than selective inhibition of EGFR as seen with LAP. In addition, VEGFR-TKIs are also reported to have better penetration to the cerebrospinal fluid than EGFR-TKIs (Hingorani et al. 2014; Tavassoly et al. 2020).

Striatal dopaminergic neuronal loss in HD patients leads to imbalanced neurotransmitters, leading to motor defects, cognitive impairment, and mood disturbances. Adjusting such imbalance could improve behavioural performance and reduce Glu-mediated excitotoxicity (Saad et al. 2021; Anderson et al. 2022; Sayed et al. 2020; Senousy et al. 2022). Herein, the observed motor–cognitive–psychiatric improvement after treatment with LAP or PAZ could be explained by the preservation of striatal dopaminergic neurons and the modulation of striatal neurotransmitter levels. Following 3-NP injection, striatal neurotoxicity was evident as neuronal oedema, dark degenerating neurons, diffuse gliosis, in accordance with previously published literature (Kumar et al. 2006; Suganya and Sumathi 2017; Saad et al. 2021; Sayed et al. 2020). Moreover, a pronounced decline in ACh, NE, DA, and 5-HT besides an elevation in Glu levels was observed, in agreement with previous studies (Suganya and Sumathi 2017; Holland et al. 2021). Treatment with LAP or PAZO mitigated these histopathological anomalies, increased ACh, NE, DA, 5-HT, and decreased Glu levels, consistent with earlier reports (Liu et al. 2011; Yang et al. 2015). Of note, the effect of PAZO was more pronounced than LAP as it significantly increased NE and DA and decreased Glu compared to LAP treatment, potentially due to superior blood–brain barrier penetration (Tavassoly et al. 2020).

In the current study, striatal TH reactivity was significantly declined following 3-NP administration as evidenced by reduced staining intensity and the lack of positively stained cells, consistent with the findings of Saad et al. 2021(Saad et al. 2021). Conversely, treatment with LAP or PAZO substantially elevated striatal TH-immunoreactive cells. Requejo et al. (2018) and Tavassoly et al. (2020) described that EGFR or VEGFR inhibition increased TH levels and prevented further neuronal loss in experimental models of brain injury and PD.

In the current investigation, rats in the 3-NP group exhibited a pronounced inflammatory status in striatal tissues, manifested by increased TNF-α and NF-κB levels alongside overexpression of their receptors, consistent with previous studies (Attia et al. 2019; Li et al. 2020; El-Sahar et al. 2020). Moreover, there was a marked increase in GFAP immunostaining in striatal tissues, in line with the findings of Sayed et al. (2020). Treatment with LAP or PAZO mitigated the observed neuroinflammation by suppressing the above-mentioned parameters. These findings are in harmony with previous studies describing the anti-inflammatory effects of various TKIs (Chen et al. 2019; Ryu et al. 2019; Li et al. 2021). The anti-inflammatory action of both drugs LAP and PAZO is mediated, at least in part, via suppression of the PI3K/AKT cascade, which leads to reduced phosphorylation of IκB-α, a key regulator of NF-κB activation (Ma et al. 2013; Zhou et al. 2020). Moreover, PAZO alleviated neuroinflammation and protected dopaminergic neurons in LPS-mouse model of PD and hippocampal neurons in d‑galactose/ovariectomized rat model of AD via suppressing MEK4-JNK-AP-1 and RIPK1/RIPK3/MLKL necroptosis signalling pathways, respectively (Sun et al. 2023; Abdelhady et al. 2023). Notably, in the current study, PAZO administration resulted in pronounced reduction in TNF-α and NF-κB levels, gene expression of TNF-α and NF-κB receptors in addition to GFAP immunoreactivity compared to LAP treatment.

Disruption of the PI3K/AKT/m-Tor signalling pathway is associated with defective autophagy, which is implicated in the pathogenesis of HD (Sayed et al. 2020). In the present work, rats intoxicated with 3-NP exhibited elevated striatal PI3K and AKT and reduced m-Tor levels. This is consistent with the findings of Mustafa et al. (2021) and Senousy et al. (2022). Although PI3K and AKT are canonical upstream activators of m-Tor, previous studies have demonstrated that under certain pathological conditions, these signalling components could become uncoupled. Recently, it was reported that m-Tor activity can be maintained or suppressed independently of AKT signalling in potent tumour suppressor phosphatase and tensin (PTEN)-deficient prostate cancer models, due to compensatory feedback loops or stress-induced regulatory mechanisms (Mao et al. 2025). In HD, oxidative stress and mitochondrial dysfunction may activate PI3K/AKT as a survival response, whilst concurrently activating AMPK or other stress pathways that inhibit m-Tor signalling to conserve cellular energy. This decoupling may contribute to impaired autophagy regulation (Meng et al. 2025). In the current work, 3-NP administration also raised striatal calpain-2 level, which has been shown to break down autophagy-related proteins. Correspondingly, striatal ULK-1, Beclin-1, and LC3-II levels were markedly declined. Such a decline in the autophagy-related proteins elucidates the involvement of defective autophagy in the 3-NP-induced neurotoxicity as reported previously (El-Sahar et al. 2020; Saad et al. 2021). Furthermore, a previous in vitro study reported that 3-NP intoxication increased calpain activity, contributing to the necrotic morphology and accelerated neuronal death (Pang et al. 2003). Noteworthy, increased calpain-2 leads to PTEN downregulation reducing the PTEN inhibitory effect on the PI3K/AKT pathway (Matsumoto et al. 2016; Panwar et al. 2023). However, high calpain-2 level could indirectly inactivate the AKT downstream pathway, explaining the observed decrease in m-Tor despite enhanced PI3K/AKT (Morgan et al. 2009; Briz et al. 2013; Butler et al. 2017). Moreover, up-regulation of calpain-2 deactivates ULK-1 and hence inhibits the stimulation of Beclin-1 after 3-NP intoxication, resulting in suppression of downstream autophagic processes (Weber et al. 2019).

By contrast, administration of LAP or PAZO markedly decreased PI3K, AKT, and calpain-2 levels whilst increasing m-Tor, ULK-1, Beclin-1, and LC3-II levels in the present study. Similarly, the TKIs dasatinib and neratinib inhibited the PI3K/AKT signalling (Dent et al. 2020). Also, PAZO and sunitinib administration increased m-Tor activity in the metastatic clear cell renal cell carcinoma (Jeong et al. 2023). As m-Tor regulates ULK-1 activation, such an increase in m-Tor level subsequently activates the ULK-1/Beclin-1/LC3-II pathway. Moreover, it was reported that autophagy activation could be achieved by inhibiting calpains in various neurodegenerative diseases (Weber et al. 2019). Notably, the effect of PAZO was more pronounced than LAP. This may be due to its broader kinase inhibition profile (VEGFR, PDFGR, FGR and c-Kit), promoting complete autophagic flux via activation of LC3-II and ERK1/2 pathways. However, LAP, acting on EGFR and HER-2, induced a partial autophagic response with p62/SQSTM1 accumulation suggesting an incomplete autophagic flux with impaired autophagosome degradation (Elshazly et al. 2024).

It is noteworthy that the human equivalent dose (HED) corresponding to the administration of 100 mg/kg LAP or PAZO in rats is 16.22 mg/kg for humans, equating to approximately 973 mg daily for a 60 kg adult, based on established interspecies dose conversion methods (Jacob et al. 2022). When compared with approved clinical doses, the standard daily dose of LAP is approximately 1250 mg, indicating that the estimated HED of 973 mg is of the same order of magnitude (Zhou et al. 2021). Similarly, the recommended clinical dose of PAZO is 800 mg once daily, which is also comparable to the HED estimate (Wu et al. 2022). Notably, PAZO monotherapy at this dosing has demonstrated a manageable toxicity profile, most commonly including diarrhoea, hypertension, and fatigue (Mederle et al. 2024). By contrast, LeVee et al. (2025) demonstrated that LAP monotherapy at comparable doses is rarely associated with cardiac adverse events. These findings highlight the need for future well-designed clinical trials to validate the calculated HEDs, refine dosing regimens, and comprehensively assess the safety and therapeutic efficacy of LAP and PAZO across diverse patient populations.

In conclusion, the present investigation provides evidence that TKIs, LAP and PAZO effectively ameliorated the 3-NP-induced HD rat model, with a pronounced effect for PAZO. Their neuroprotective effects could be attributed to the mitigation of neurodegeneration and neuroinflammation along with restoration of the PI3K/AKT/m-Tor-mediated autophagy cascade. The observed crosstalk between this cascade and calpain-2 reveals the role of calpain-2 in moderating autophagy and, consequently, neuronal survival in HD. These findings support the potential repurposing of TKIs as therapeutic agents for HD, offering prospects for improved quality of life and reduced healthcare burden.

## Data Availability

The datasets generated during and/or analyzed during the current study are available from the corresponding author on reasonable request.
